# Changing Cancer Trends in District Dir, Pakistan: Epidemiological Insights From a 10-Year Hospital-Based Study

**DOI:** 10.7759/cureus.62944

**Published:** 2024-06-23

**Authors:** Sunnia Shah, Anusha Azhar, Saud Azhar, Maaz Khan

**Affiliations:** 1 Community Medicine, Khyber Medical College, Peshawar, PAK; 2 General Medicine, Hayatabad Medical Complex Peshawar, Peshawar, PAK; 3 Oncology, Khyber Medical College, Peshawar, PAK

**Keywords:** pakistan, public health, epidemiology, cancer prevalence, cancer incidence, preventive oncology, cancer

## Abstract

Background

More alarming than the rise of cancer globally is its discreet changing profiles over the years. According to our knowledge, no new studies on cancer have taken place in Dir since 2004. Hence, we aimed to provide and analyze the cancer trends in district Dir, Malakand division, Khyber Pakhtunkhwa (KPK), regarding its prevalence and incidence, and compare it nationally and internationally.

Methods

A retrospective study was conducted by collecting data from 2647 clinically diagnosed cancer patients of all ages in district Dir, between the years 2008 and 2017, from the Institute of Radiotherapy and Nuclear Medicine (IRNUM), Peshawar. Statistical analysis was performed using SPSS version 20 and presented using different tables and figures.

Results

Out of the total patients, 52.7% were male and 47.3% were female. The most common types of cancers in both genders combined were breast (9.0%), acute lymphocytic leukemia (ALL) (6.0%), skin (5.7%), non-Hodgkin Lymphoma (NHL) (5.6%), and brain tumor (5.2%). The age-standardized incidence rate (ASIR) in males in 2017 was peaking in the age group 60-69 (2707.2) with the most prevalent cancer being NHL (7.7%). In females, ASIR was highest in the age group 30-39 (2500.8) with the majority having breast cancer (18.1%), and in children, ALL (30.9%) was most prevalent. Incidence was highest in 2014 with a staggering 15 cases/100,000 population. Cancer prevalence in females aged 50 and below was significantly higher (p<0.001) compared to males.

Conclusion

Our study highlights that cancer profiles in Dir in the past two decades have changed with certain results non-conforming to global and regional trends. A follow-up research should be carried out to further ascertain and analyze these diverging results in hopes of drawing a more concrete conclusion from these findings.

## Introduction

Cancer is an umbrella term for many diseases that can start in almost any organ or tissue of the body when abnormal cells grow uncontrollably, go beyond their usual boundaries to invade adjoining parts of the body, and/or spread to other organs [1].

Cancer is one of the deadliest diseases mushrooming across all continents. According to a study conducted on all-cause mortality, in 2019 by WHO, trachea, bronchus, and lung cancer ranks 6th worldwide [2]. In upper-middle-income countries, lung cancer ranks 4th, and stomach cancer occupies 9th position among the top 10 most common causes of death, portraying the highly fatal nature of this disease. A major concern is not only cancer’s lethality but also the alarming pace it is growing worldwide. In the past 45 years from 1975 to 2020 cancer has been predicted to rise by more than 20% in the United States alone [3].

The distribution of cancer was also associated with different socioeconomic groups. Qualitative research on 91 countries in Europe from 2000 to 2019 uncovered that adults belonging to low socioeconomic status (SES) had the highest relative risks for lung, head and neck, stomach, and cervix cancer. Conversely, high SES was linked with an increased risk of thyroid, breast, prostate, and skin cancers [4].

Cancer epidemiology in South Asia - consisting of Pakistan, India, Sri Lanka, Bangladesh, Nepal, and Bhutan - revealed similarities in the prevalence patterns with oral and lung cancers being generally number one or number two in most countries in males. High numbers of pharyngeal and laryngeal cancers were also observed, with prostate cancer mainly appearing in developed cities. Except for Pakistan which had oral cancer followed by breast cancer, all other South Asian countries had cervical cancer becoming the second most common type of cancer after breast [5].

In Pakistan, a study conducted by the Health Research Institute from 2015 to 2016 stated that cancer is a major public health problem where about 148,000 new cancer cases are diagnosed and about 100,000 die due to cancer annually. Unfortunately, its deadly nature is not the only feature Pakistan has to combat but also curbs the decades-long proliferation as it is estimated that the total trend of combined breast and lung cancer cases in Pakistan will surge from an annual 43,837 in 2018 to 81,789 by 2040 [6].

In Pakistan, women are majorly affected by breast cancer (28.7%) and males by lip and oral cavity cancer (12.9%), in different provinces [7]. A descriptive study led by the Breast Unit, Department of Surgery, Liaquat National Hospital Karachi, Pakistan from 1994 to 2016 revealed that in the respective period 10,018 patients with breast cancer were registered, many being in younger age groups as compared to the West as well as being in the advanced stages, reflecting that breast cancer is starting in early stages in Pakistan with a shortage of screening facilities [8].

Although the scarcity of data on cancer trends in the province of Khyber Pakhtunkhwa (KPK) is proving to be a challenge, a hospital-based study in the District Dir of KPK province, in 2008, presented the annual incidence rates of cancer from 2000-2004, in which 62% were male and 38% were female, further highlighting the increased incidence of cancer in the district [9], while also accentuating the fact that the most affected occupational group in the district was that of farmers followed by housewives [10]. Although another research investigation designed to examine and analyze the prevalence of cancer in residents of District Bannu, KPK, in 2005-09, revealed that the number of cancer patients decreased per annum [11], proving that these local trends need further studying.

Our primary objective is to determine and analyze the 10-year cancer trends of district Dir with regard to its prevalence and incidence. Another aim of this study is to compare this newer set of data with the previous study conducted in Dir, with both international and national figures, and discuss the possible risk factors of different types of cancers in Dir.

## Materials and methods

A retrospective record-based study was conducted by collecting secondary data from patients’ records from the major cancer hospital of KPK which is the Institute of Radiotherapy and Nuclear Medicine (IRNUM), Peshawar. The ethical approval was taken both from the hospital and Institutional Research and Ethical Review Board (IREB) (Approval number: 344/DME/KMC).

Our study setting was IRNUM hospital, as it is the oldest, largest, and the sole government-based cancer hospital of KPK province and caters to a large population of district Dir. All the data was collected in secondary form, from patients’ past medical records, from the hospital management information system.

Inclusion/exclusion criteria

All the patients who were diagnosed with cancer, admitted, and/or treated in IRNUM hospital, Peshawar in the years 2008-2017, and whose data was available in the records of the hospital, were included in the study. The population included both males and females, of all ages, belonging to district Dir (Upper and Lower). Patients whose data was missing from the records were mixed up and not categorized properly were excluded.

The study variables included year of diagnosis of cancer, age of the patient, gender of the patient, district to which the patient belonged, site of the cancer, stage of the cancer, grade of the cancer, treatment given, tumor status of the cancer, necrosis, and metastasis status of the cancer. For certain variables, the following data was missing due to either non-applicability or incomplete data entry in subsets: grade of cancer (n = 1965, 74%), stage of cancer (n = 421, 15.9%), tumor status of the cancer (n = 421, 15.9%), necrosis (n = 338, 12.8%) and metastasis status of the cancer (n = 349, 13.2%). Hence, this data was excluded from the analysis.

The data was analyzed using Statistical Package for Social Sciences (SPSS) version 20. To test the proportions of cancer patients with age and gender groups chi-square test was performed. Age-standardized incidence rate (ASIR) was calculated using its formula. The data for the gender and age-wise population of district Dir (Upper and Lower) and Pakistan was taken from the Pakistan Bureau of Statistics and put into the formula [12]. The incidence and prevalence of cancer in relation to different age and sex groups were also calculated. Annual percentage change (APC) was calculated by finding the difference between two incidence values of two subsequent years and dividing it by the incidence value of the preceding year. The values are expressed in percentage form. The average annual percentage change (AAPC) was also calculated. All data has been presented in the form of tables and figures.

## Results

Out of the total 2647 patients considered for analysis from the districts of Upper Dir and Lower Dir, 1396 (52.7%) were male, and 1251 (47.3%) were female. Most of the patients recorded belonged to the district of Lower Dir (n = 2338, 88.3%) in all these 10 years (2008-2017).

**Figure 1:**
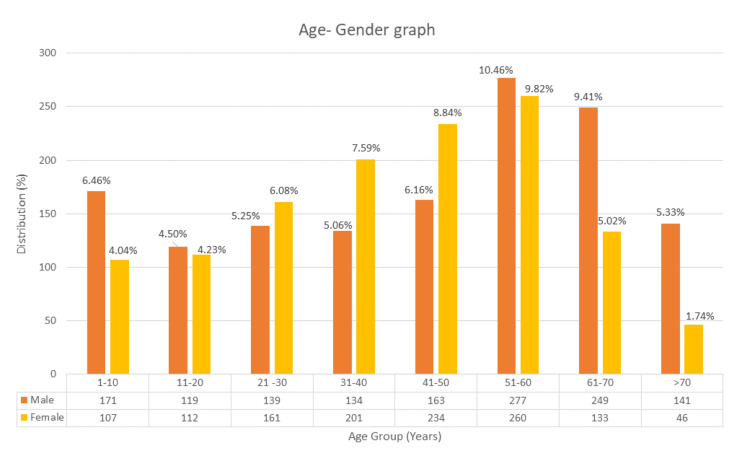
Age-gender distribution of cancer patients in Dir. Each gender in every age group is presented with its frequency and percentage with regard to the total population.

The most common cancers in both genders combined were revealed to be breast cancer (n=239, 9.0%), acute lymphocytic leukemia (ALL) (n = 158, 6.0%), skin cancer (n = 150, 5.7%), non-Hodgkin’s lymphoma (NHL) (n = 148, 5.6%), and brain tumor (n = 138, 5.2%) as shown in Table 1. The age-wise distribution of the top 10 most prevalent types of cancer is also shown in Table 1. The age- and gender-wise distribution of cancer is presented in Figure 1.

**Table 1:** Age-wise distribution of the top 10 most prevalent cancers in both genders combined in Dir district from 2008 to 2017. The age group of 41-50 shows the highest frequency of breast cancer. ALL was found to be most prevalent in patients under the age of 20. Skin cancer assumes command in the age group of 61-70 while NHL leads in the age group of 51-60. Brain tumors were most recurrent in the age group of 31-40 years old. AML: acute myelogenous leukemia; NHL: non-Hodgkin lymphoma; ALL: acute lymphocytic leukemia

Site of Cancer	Age Group								Total
	1-10	11-20	21-30	31-40	41-50	51-60	61-70	>70	N (%)
Breast	0	1	40	55	61	53	23	6	239 (9.0)
ALL	86	42	16	8	2	2	1	1	158 (6.0)
Skin	2	1	8	9	22	32	44	32	150 (5.7)
NHL	16	20	10	15	18	35	23	11	148 (5.6)
Brain tumor	20	19	20	24	15	21	17	2	138 (5.2)
Metastatic carcinoma	4	5	14	20	25	20	12	7	107 (4.0)
Ovary	7	14	15	9	23	16	8	6	98 (3.7)
AML	22	16	12	10	9	16	5	5	95 (3.6)
Prostate	5	3	4	3	11	17	24	17	84 (3.2)
Rectum	4	4	14	17	15	13	7	6	80 (3.0)

The cancer incidence for the year 2017 for males and females were 10.35 and 8.66, respectively. The age-standardized incidence rates (ASIR) for each 10-year age group (0-70+), in 2017, are mentioned in Table 2. The incidence rates were computed by age group and sex and presented per 100,000 population. The combined ASIR for males was 12.75 and for females 10.62.

**Table 2:** Age-standardized incidence rates (ASIR) based on age and sex distribution per 100,000 population for district Dir in 2017.

	No. of Cancer Cases		Person-Years at Risk		Standardized Population of Pakistan		ASIR	
Age Group	M	F	M	F	M	F	M	F
0-9	9	6	429,041	404,122	30,586,849	28,602,459	641.62	424.66
10-19	12	13	298,344	284,273	24,042,009	21849,530	967.02	999.19
20-29	13	14	188,521	204,273	17,181,392	17709,564	1184.79	1213.74
30-39	12	17	118,618	89,470	13,033,676	13,161,660	1318.55	2500.82
40-49	13	20	77,308	80,268	89,08,220	86,46,944	1497.99	2514.52
50-59	27	20	47,486	47,929	62,90,833	56,27,636	3576.90	2348.32
60-69	24	8	33,733	33,988	38,05,074	34,57,413	2707.19	813.80
70+	16	3	23,811	22,475	24,70,167	22,89,426	1659.85	305.60
Total	126	101	12,16,862	11,66,798	106,318,220	101,344,632	13553.92	10760.64

The top three most prevalent cancers in men were NHL (n = 107, 7.7%) and ALL (n = 106, 7.6%) followed by brain tumor (n = 92, 6.6%). In females, the most common cancer was found to be breast (n = 227, 18.1%), followed by ovary (n = 81, 6.5%) and finally skin cancer (n = 65, 5.2%).

**Figure 2:**
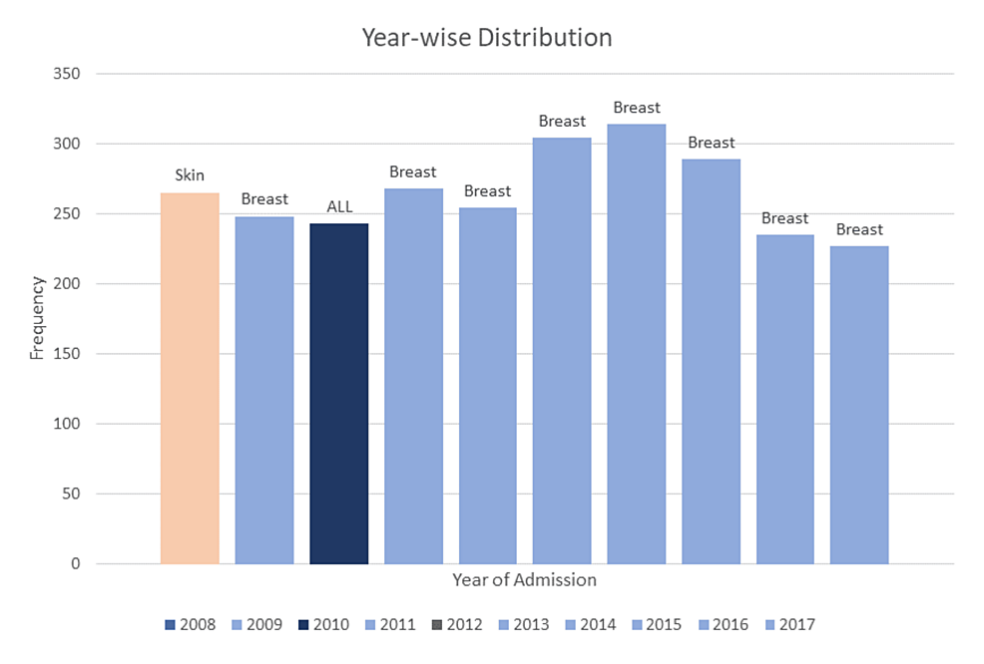
Year-wise distribution of cancer cases reported alongside the distribution of most prevalent cancer of every year from 2008 to 2017. In 2008, the highest number of cases reported were of skin cancer (n = 24, 9.1%), followed by breast cancer in 2009 (n = 28, 11.3%), followed by ALL in 2010 (n = 20, 8.2%), after which breast cancer remained at the top for the next 7 years with an average of 27 cases per year.

The most repetitive cancer year-on-year in the entire population of Dir was breast cancer (Figure 2). The highest number of patients was recorded in 2014 (n = 314, 11.9%) while the least number of patients recorded was in 2017 (n = 227, 8.6%).

**Figure 3:**
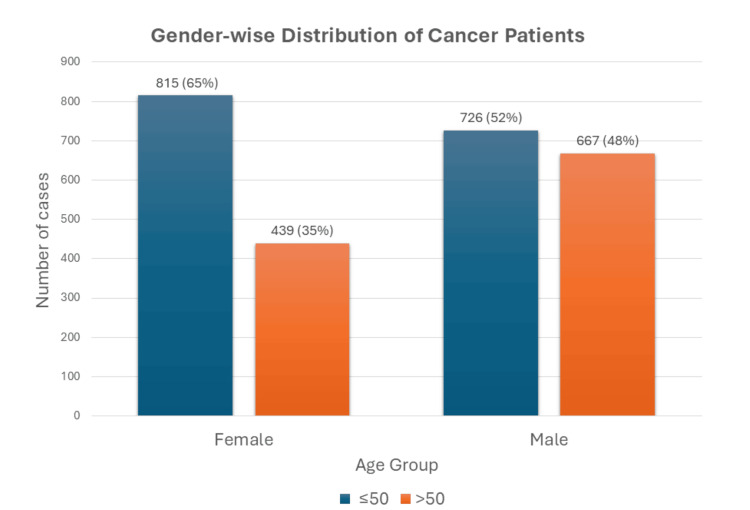
Gender-wise distribution of all cancer patients in age groups above and below 50 years. Chi-square= 44.70, p-value= 0.0001; odds ratio= 1.71 with 95% CI (1.50, 2.00).

Eighty one percent of the patients were treated with chemotherapy and radiation. Overall analysis showed that cancer was significantly higher in females aged ≤ 50 (n = 815, 65.0%) as compared to those > 50 years (n = 439, 35.0%). In males, the difference in the number of cancer cases in patients >50 years (n = 667, 47.9%) and those ≤ 50 years (n = 726, 52.1%) was minimal. A chi-square test of independence was performed to examine the relation between gender and age groups (Figure 3). The relation between these variables was significant, X2 (1, N = 2647) = 44.97, p =.0001. Women below the age of 50 were much more likely than men to present with cancer. The odds ratio was 1.71 with a 95% confidence interval (CI) (1.46, 2.00).

The annual incident rates, annual percentage change (APC) and average annual percentage change (AAPC) from 2008 to 2017 are presented below in Table 3.

**Table 3:** Cancer incidence and APC from 2008 to 2017. *Incidence is per 100,000 population; ⇴ total number of patients registered in IRNUM from 2008 to 2017; ⇞ mean incidence/year. AAPC: average annual percentage change

Year	Patients (n)	Population (n)	Incidence*	Annual Percentage Change (APC)
2008	265	17,54,154	15.12	-
2009	248	18,06,245	13.73	-9.2%
2010	243	18,60,244	13.06	-4.9%
2011	268	19,15,389	13.99	7.1%
2012	254	19,82,390	12.81	-8.4%
2013	304	20,51,498	14.82	15.7%
2014	314	20,98,297	14.96	0.9%
2015	289	21,98,422	13.15	-12.1%
2016	235	22,68,293	10.36	-21.2%
2017	227	23,50,024	9.66	-6.8%
	2647^⇴^		13.17^⇞^	-1.78% (AAPC)

The incidence rates (cases/1,000,000) of the top five most common cancers of the year 2017 were also calculated. Breast cancer led with an annual incidence of 8.5 cases, followed closely by NHL with 7.6 cases, then skin cancer with an incidence rate of 5.5, for fourth place stomach with 4.7 cases, and in last place tied ALL, rectum, bone, and urinary bladder cancer with an annual incidence of 4.3 cases.

## Discussion

Cancer has always been a major public health concern, even more so now with increasing population rates [6]. Our study was conducted mainly to shed light on cancer incidence and burden in a more geographically remote area like Dir where cancer trends are quite different when compared to the global and national rankings of cancer.

Out of the total cancer patients from 2008 to 2017, 52.7% were male and 47.3% female compared to the last study conducted there which only had 38% females reported to have cancer [9]. This finding could be related to the increased literacy rates in KPK province with a 24.6% increase in the span of 14 years from 1998 to 2012 [13], which in turn led to more awareness and hence more reporting of malignancies. Not surprising, however, was that 88.3% of patients belonged to Lower Dir, as its population is two-thirds of the total district [14].

The data collected revealed that the most prevalent type of cancer in the population was breast cancer (n = 239, 9.0%) which could be attributed to the low educational rates, lack of awareness, limited screening tests, sedentary lifestyles, and high fatty diet among various other reasons. This finding conformed with multiple other studies, both on an international and national level. A study by WHO in 2020 showed breast cancer prevalence of 14.5% out of all cancers [7] whereas the other one in Karachi showed similar results in 2014 [15]. Breast cancer was found to be most prevalent in the age group of 41-50 which can be associated with the surplus body weight and high body mass index in females in that region [16,17]. Another reason for such high numbers of these cases could be due to the radioactive elements found in the Hindu Kush Mountain range-which borders the northwest region of Dir. The radiation has been associated with increased breast cancer prevalence in the Malakand division [18].

The second most common cancer was ALL with a total of 158 (6.0%) cases, mainly dominating the first two decades of life. In the age group of 1-10 years, it also holds the highest number of cases (n = 86) of any type of cancer reported in any age group. ALL is responsible for 30% of all cancers in American children under 15 years of age [19] and 19.8% of cancers in Pakistani children [20]. Such a towering number of cases can be co-related with the higher than acceptable concentrations of radioactive soil found in the northern regions of Pakistan, compared to the south [21]. Generations of exposure to such radiation, subsequent accumulation of germline mutations, and the ultimate manifestation of these insults in the form of ALL could allude to these findings. Studies have also shown that both the annual absorbed cosmic radiation as well as the lifetime excess cancer risk in regions near Dir exceeded the average world values [22]. These results warrant targeted interventions in Dir to diminish if not halt the growing spread of this burden.

Skin cancer held third place, with a total of 150 (5.7%) cases. This position when compared with other regions is a bit higher as worldwide skin cancer ranks fifth (5.8%), while according to the Punjab Cancer Registry, it ranks eighth and ninth most common cancer in females and males respectively in 2017. Karachi Cancer Registry (KCR) also reported skin cancer among the top eight cancers in females in Karachi [23]. This jump to a higher ranking in our study could foremost be associated with dangerous UV radiation due to the high altitude of Dir at just under 5000 ft and excessive sun exposure due to occupational needs [24]. Most of the cases were found to be in the age group of 61-70, which is the most common age group for manifestation of melanoma.

According to our research, a total of 148 (5.6%) cases of NHL were reported. This result although not adhering to the conventional global cancer triad [25] - consisting of breast, lung, and colorectal cancer - is consistent with the general trend of increasing cases of NHL as we move from south to north in Pakistan as well as the high incidence rates especially found in KPK [26,27]. Interaction of environmental factors, exposure to agricultural chemicals, and genetic predispositions can partially explain the unique trends although further prospective studies ought to be conducted to help better assess the underlying reasons and formulate interceding strategies accordingly.

Despite the somewhat predictable common contender for the first four places, the cancer ranking fifth place is noteworthy; brain tumor (n=138, 5.2%) which isn't included in the top 15 most common cancers of the world according to a study conducted in 2020 by World Cancer Research Fund International [28], nor is it making the top five of the most prevalent cancers in Pakistan according to the data compiled from cancer registry from 1994 to 2021 [19]. A potential cause of the high emergence of this tumor could be related to the ionization radiation as a study published in 2021 portrayed that 43.99% of the total young males who had primary malignant brain tumors (PMBTs) had a history of living at high altitude at some point of time due to occupational requisites [29]. The incidence in the study was highest in the age group of 30-39-year-olds, in males which matches the findings in Dir. However, the very high percentage of this cancer cannot be explained by environmental factors alone hence further epidemiological studies are required to unearth any other causes including genetic susceptibilities, atopic diseases, correlation with gender, etc.

Our study showed ALL, acute myelogenous leukemia (AML), and brain tumors to be more prevalent in children which is consistent with other literature [30]. Many of the cancers shown in Table 1 were diagnosed after 40 years of age. Lack of education and awareness programs, paucity of screening tests, reluctance, and hesitance of rural people to get regular check-ups, and increased probability of accumulated genetic insults in advanced ages can shed some light on this finding [31].

The prevalence of cancer in females aged 50 and below was significantly higher when compared to males. This finding can be associated with the fact that the most prevalent cancer in women was breast cancer which was predominantly found in the age group of 41-50 years.

The number of cancer cases reported each year showed a general upward trend, being highest in 2014 and lowest in 2017. According to the previous study conducted in Dir [9] as well as the generalized increase in cancer rates both worldwide and nationwide [25] it is surprising to note the downfall of cases after 2014.

Unfortunately, due to the start of the COVID-19 pandemic, further data could not be retrieved. It is plausible that the reason behind diminishing cancer cases in Dir could be applied on a national level, making the unveiling of this aberration a top-priority motive. The data we received included patients whose information was missing or mixed up; such patients were excluded from our analysis. Limitations in our study include cancer patients of Dir referring to hospitals in other provinces or being readmitted in later years, however as IRNUM hospital is the exclusive and closest cancer treatment facility in the region, selection bias is minimized.

## Conclusions

This study sheds light on the cancer burden in Dir District, Khyber Pakhtunkhwa, Pakistan, from 2008 to 2017. Key findings reveal cancer trends non-conforming to the previous regional as well as international cancer profiles. Although men in Dir are more likely to get cancer, there is a higher prevalence of cancer in females under the age of 50. ALL dominates in the pediatric population, NHL in males and breast cancer in females. Skin and brain tumors are aberrantly high in this region compared to other countries. Overall, the cancer incidence over the span of 10 years has slightly decreased. It is recommended that within a few years’ time another study spanning over the next 10 years be conducted to note the latest changing cancer profiles and compare it with recent studies. There is an urgent need for increased awareness and improved access to screening and treatment facilities to mitigate the growing cancer burden in this region.
